# Antioxidants positively regulate obesity dependent circRNAs - sperm quality - functional axis

**DOI:** 10.3389/fendo.2023.1290971

**Published:** 2023-12-11

**Authors:** Vincenza Grazia Mele, Teresa Chioccarelli, Rosario Finamore, Antonella D’Agostino, Maria d’Agostino, Donatella Cimini, Monica Mattia, Veronica Porreca, Andrea Maria Giori, Silvia Fasano, Gilda Cobellis, Chiara Schiraldi, Rosanna Chianese, Francesco Manfrevola

**Affiliations:** ^1^ Department of Experimental Medicine, University of Campania “Luigi Vanvitelli”, Naples, Italy; ^2^ Department of Sciences and Environmental, Biological and Pharmaceutical Technologies, University of Campania “Luigi Vanvitelli”, Caserta, Italy; ^3^ R&D Department, Institut Biochimique SA (IBSA) Italia Srl, Lodi, Italy

**Keywords:** antioxidant agents, backsplicing, circRNAs, male infertility, obesity, oxidative stress, spermatozoa

## Abstract

Obesity is a pathophysiological condition, dependent on body fat accumulation, that progressively induces systemic oxidative stress/inflammation leading to a set of associated clinical manifestations, including male infertility. CircRNAs, covalently closed RNA molecules, are key regulators of sperm quality. Recently, we have characterized a complete profile of high-fat diet (HFD) spermatic circRNA cargo, predicting paternal circRNA dependent networks (ceRNETs), potentially involved in sperm oxidative stress and motility anomalies. In the current work, using HFD C57BL6/J male mice, orally treated with a mix of bioactive molecules (vitamin C; vitamin B12; vitamin E; selenium-L-methionine; glutathione-GSH) for 4 weeks, a reversion of HFD phenotype was observed. In addition, the functional action of the proposed formulations on circRNA biogenesis was evaluated by assessing the endogenous spermatic FUS-dependent backsplicing machinery and related circRNA cargo. After that, spermatic viability and motility were also analyzed. Paternal ceRNETs, potentially involved in oxidative stress regulation and sperm motility defects, were identified and used to suggest that the beneficial action of the food supplements here conveniently formulated on sperm motility was likely due to the recovery of circRNA profile. Such a hypothesis was, then, verified by an *in vitro* assay.

## Introduction

Obesity is a pathophysiological condition, affecting more than a third of the population, dependent on the accumulation in body fat (1). The resulting chronic inflammatory state is tightly responsible for biochemical anomalies, including systemic oxidative stress/inflammation and insulin resistance that, in turn, induce a set of associated clinical manifestations such as liver steatosis, cardiovascular diseases, type 2 diabetes mellitus and neurodegeneration (1–4).

Obesity onset involves several risk factors, including: i) genetic predisposition linked to single nucleotide polymorphisms (SNPs) and epigenetic inheritance (5); ii) psychological factors; iii) sedentary lifestyle (5–7).

In this scenario, there is a growing concern regarding the effects of obesity on male fertility. Under the guidance of inflammatory cytokines and adipokines, metabolic dysregulation impacts on spermatogenesis, by disrupting steroidogenesis and inducing germ cell apoptosis (8, 9). A strong effect has also been described on sperm morphological and functional parameters, especially on head morphology, concentration, viability and motility (9–11). In particular, an increased prevalence of asthenozoospermia has been observed in obese males (12), as a result of oxidative stress dependent mitochondrial dysfunctions that negatively impact on sperm motility (13–16). Considering that unbalanced oxidative pathways underlie the biochemical changes responsible for obesity dependent clinical anomalies, a growing interest on bioactive ingredients/antioxidant-based food supplements, as possible therapeutic approach able to counteract obesity complications, is emerging. In this context, beneficial effects of vitamin C (ascorbic acid), in terms of adipocyte lipolysis modulation, inflammatory response reduction and leptin secretion inhibition, have been reported (17, 18). Similarly, vitamin E has been described as potential antioxidant and anti-obesity factor able to reduce collagen deposition in visceral adipose tissue of high fat-diet (HFD) mice, inducing insulin sensitivity improvement (19, 20). Vitamin B12 deficiency has also been associated with obesity condition (21). Interestingly, L-selenomethionine diet supplementation has been associated with an enhanced adipose tissue beiging process in HFD-induced obesity (22, 23), while oral glutathione (GSH) supplementation improves insulin sensitivity in obese subjects (24). Growing evidences have highlighted how the therapeutic application of antioxidant agents in obesity condition positively correlates with improved sperm parameters. In details, vitamin C supplementation in infertile men enhances sperm count, motility and morphology parameters (25, 26), while vitamin E *in vitro* treatment on spermatozoa (SPZ) collected from asthenoteratozoospermic men improves motility parameters protecting SPZ from oxidative stress (27).

CircRNAs, covalently closed RNA molecules produced by backsplicing (28, 29), are acquiring a prominent role as key players in the regulation of sperm quality, as they appear differentially expressed in relation to morphological and functional sperm parameters, as well as to pathological conditions, including asthenozoospermia (30–32). In particular, the endogenous skill to circularize mRNAs in circRNA counterpart, highlighted in both murine and human SPZ, seems to be tightly correlated with the intrinsic sperm quality state, thus electing circRNAs as potential biomarkers of sperm quality (33, 34). We have previously profiled a spermatic circRNA cargo in HFD murine SPZ and demonstrated a dysregulation in the endogenous backsplicing machinery dependent on Fused protein in Sarcoma (FUS), the main RNA binding protein (RBP) orchestrating circRNA biogenesis (35). In addition, we have predicted circRNA dependent networks (ceRNETs) potentially involved in sperm oxidative stress and motility anomalies occurring in HFD model.

Considering the ameliorative effects on fat body mass and sperm quality parameters after antioxidant based treatments, here we shed light on the potential interplay among circRNAs and sperm quality parameters under antioxidant agent regulation. To achieve this goal, HFD male mice were orally treated with a food supplementation (FS) of integrator mix (vitamin C; GSH; vitamin E; selenium-L-methionine; vitamin B12) for 4 weeks and sacrificed to assess morphological, functional and molecular sperm parameters. An increase in sperm motility and viability was observed following FS treatment. About that, a reduction of 4-hydroxynonenal (4HNE), a lipid peroxidation marker typically chosen to assess oxidative stress in cells, also occurred following FS treatment.

Interestingly a set of spermatic circRNAs, typically up-regulated in HFD SPZ, was restored to physiological values, demonstrating the beneficial action of antioxidant agents on backsplicing modulation. Such an effect was molecularly dependent on spermatic FUS restoration in terms of protein content and subcellular distribution. The *in vitro* induction of oxidative stress in SPZ definitively demonstrates the existence of a functional axis linking oxidative stress - circRNAs - sperm quality.

## Material and methods

### Animals, diet and food supplement

*Mus musculus* C57BL6/J mice were purchased from ENVIGO (Udine, Italy). The control “normal fat” diet (CTRL) contained 63% Carbohydrates, 22% Protein, 15% Fat, while HFD contained 18% Carbohydrates, 22% Protein, 60% Fat; both diets were specifically designed, developed and administered by ENVIGO Service.

The FS used for the animal treatment was composed of: fish oil (42.29%), vitamin C (24.58%), vitamin E (22.19%), glutathione (7.41%), vitamin B12 (1.47%), L-selenomethionine (0.06%). Given the composition, FS was dissolved in an oil solution and daily provided to the animals as 32.9 total mg/average body weight (see **Table 1**). The ingredients were obtained from IBSA Farmaceutici - R&D department (Lodi, Italy) in the framework of *Incube* project, with purity and characteristics suitable for human use. In fact they are already part of a FS formulation approved by the Istituto Superiore di Sanità (ISS) in agreement with the current regulatory demand.

**Table 1:** The components and their percentage in the FS.

Day formulations (dose 32.9mg)	mg component/tot mg (dose in %)
70% fish oil	44.29
Vitamin C	24.58
Vitamin E (from Alpha-tacopherol)	22.19
Glutathione	7.41
Vitamin B12 0.1%	1.47
L-selenomethionine	0.06

The total daily dose for each mouse was 32.9 mg.

### Experimental design

Eight-week-old male mice were individually caged and randomly divided in two groups, exposed to a normal (CTRL group; n=12) or high fat (HFD group; n=24) diet for 12 weeks. We chose to start the experimental feeding at 8 weeks and up to 20 weeks of age, to reproduce the increase of body weight and fat mass of an established model of diet-induced obesity (36). The service weekly recorded body weight increases and food intake to verify the experimental approach (data not shown) as previously reported (35).

After 12 weeks, all the mice were acclimated to the new environment for a week before the experiment. In detail, the mice were kept in a room with a strictly controlled temperature (22 ± 2°C), ventilation, and lighting (12-h light/dark cycles), with free access to food and water. After a week of acclimatization, HFD mice were randomly divided in two groups (n=12 per group) receiving water (daily oral gavage of 0.15 ml water for 4 weeks) or FS (daily oral gavage of 0.0329 g/day FS for 4 weeks). Conversely, CTRL mice have continued with their original diet for 4 weeks in the same controlled conditions, freely eating and drinking until the sacrifice.

The number of the enrolled animals was determined by the parameters established through the G*Power analysis (latest ver. 3.1.9.7; Heinrich-Heine-Universität Düsseldorf, Düsseldorf, Germany; http://www.gpower.hhu.de/) required to get the permission for *in vivo* experiments in Italy, suggested by the Legal Entity giving the permission.

Experiments involving animals were approved by the Italian Ministry of Education and the Italian Ministry of Health, with authorization n°405/2021-PR. Procedures involving animal care were carried out in accordance with the National Research Council’s publication Guide for Care and Use of Laboratory Animals (National of Institutes of Health Guide).

### Tissue sampling

At the end of FS treatment, body weight, length, and abdominal circumference were measured in CTRL, HFD and FS mice. After that, all animals were sacrificed and subjected to tissue collection. In detail, the animals were placed in a plexiglass chamber with 4% isoflurane (Iso-Vet, Piramal Healthcare, UK Limited) for 5 min and were sacrificed by cervical dislocation when fully sedated, as measured by a lack of heartbeat and active paw reflex. The peripheral tissues (testes and liver) were rapidly removed, weighed and stored at -80°C for molecular investigations, and/or fixed in Bouin’s solution for morphological analyses. The epididymides were dissected and used to collect *caput* and *cauda* epididymal SPZ as described below.

### Mouse sperm collection

*Caput/cauda* epididymides (from n=6 CTRL, n=6 HFD and n=6 FS) were separately immersed in PBS (pH 7.6), cut to let SPZ flow out from the ducts, and filtered throughout cheesecloth to eliminate epididymal tissue fragments. Then samples were centrifuged at 1500 x *g* for 30 min at 4°C and then SPZ pellets were incubated on ice for 30 min with Somatic Cell Lysis Buffer (SCLB; 0.1% SDS, 0.5% Triton X-100 in DEPC-H_2_O) to eliminate possible somatic cell contaminations. Following this step, SPZ were centrifuged at 800 x *g* for 15 min at 4°C and washed twice with PBS. Aliquots of total and *caput/cauda* epididymal SPZ were stored at -80°C for molecular investigations and/or dried on slides to be stored at -20°C for morphological analyses. In addition, aliquots of *cauda* SPZ were used for sperm functional analysis as above reported.

### Mouse sperm functional analysis

*Cauda* SPZ collected from CTRL, HFD and FS mice (n=6 for each experimental group) or *cauda* SPZ of CTRL mice *in vitro* treated with vehicle or H_2_O_2_ (n=6 for each experimental group), were used to investigate sperm functional parameters as viability and motility. In brief, a hemocytometer (Burker Chamber) was used to assess the number of live, motile and total SPZ, under a light microscope (Leica CTR500, Leica Microsystems Inc., Milan, Italy) by three observers. The procedure was validated by using double-blind test. Live SPZ were assessed calculating the percentage of dead/total SPZ, by using the viable dye Trypan blue reagent (Trypan Blue, 0.4% Solution, 17-942E Lonza). Motile SPZ were reported as percentage of motile/live SPZ. A minimum of 100 sperm cells was counted for each analysis.

### Mouse sperm time lapse analyses

*Cauda* SPZ were collected from CTRL, HFD and FS mice (n=6 for each experimental group) to investigate sperm motility by using time lapse video microscopy (TLVM) station and software that allow to observe and record specific areas, in real time, and thus to monitor a number of biological phenomena such as motility and eventually morphological changes (37). In the framework of these experiments, we recorded images continuously (second by second) and then we measured the displacement of a single sperm cell, analyzed in the definite time interval, by using CELL TRACKER software (OKOLAB, Italy).

Specifically, aliquots of SPZ were diluted 1:1 in PBS containing calcium and magnesium (13.6 mM NaCl; 2.68 mM KCl; 8.08 mM Na_2_HPO_4_; 18.4 mM KH_2_PO_4_; 0.9 mM CaCl_2_; 0.5 mM MgCl_2_; pH 7.6) and placed on concave slides previously pre-warmed at 37°C. Each slide was immediately engaged in the stage incubator of TLVM under controlled conditions (T=37°C, 5% CO_2_ in humidified air). For each sample, five experiments were carried out without delay time, acquiring at least fifteen images (1 img/sec). We analyzed at least 5 sperm cell/zone for each experiment.

### Total RNA preparation

Total RNA was extracted from total and *caput/cauda* SPZ or *cauda* SPZ of CTRL mice *in vitro* treated with vehicle or H_2_O_2_, by using Trizol Reagent (Invitrogen Life Technologies, Paisley, UK) following the manufacturer’s instructions. Samples were homogenized in Trizol Reagent (1 ml Trizol Reagent/mg tissue or 5-10 x 10^6^ sperm cells) and the complete dissociation of nucleoprotein complexes was allowed incubating them for 5 min at 20°C. Then 0.2 ml chloroform/ml Trizol Reagent were added to samples. A centrifugation at 12000 x *g* for 15 min at 4°C was carried out to isolate aqueous phase, that was rapidly transferred to a fresh tube. Total RNA was precipitated by mixing with isopropyl alcohol (0.5 ml/ml Trizol Reagent) and 1 µl glycogen (20 mg/ml). After centrifugation at 12000 x *g* for 10 min at 4°C, the RNA pellet was washed with 75% ethanol, centrifuged at 7500 x *g* for 10 min at 4°C and dissolved in DEPC-treated water. The quantity (ng/µl) and purity (260/280 and 260/230 ratios) of RNA samples were assessed with a NanoDrop 2000 spectrophotometer (Thermo, Waltham, MA, United States). RNA aliquots (10 µg) were treated with 2U DNase I (RNase-free DNase I, Ambion, Thermo Fisher Scientific, Massachusetts, United States) to remove genomic DNA contaminations and preserved at -80°C until the next step.

### RNA expression analysis by one-step Evagreen qRT-PCR

Evagreen qPCR Mastermix kit (Applied Biological Materials Inc., Ferndale, WA, United States) was used to perform circRNA expression analysis in sperm samples collected from CTRL, HFD and FS mice (n=6 animals for each experimental group) and in *cauda* SPZ of CTRL mice *in vitro* treated with vehicle or H_2_O_2_ (n=6 animals for each experimental group), according to the manufacturer’s instructions. A quantity of 50 ng of total RNA was used for all reaction performed in a CFX-96 Real Time PCR System (Biorad, Milano, Italy). Each assay was carried out in triplicates and a melting curve analysis for each primer pairs was performed. A negative control, without RNA, was also included. CFX Manager software (Biorad, Milano, Italy) was used for RNA expression analysis. *Cyclophilin* was used as spermatic housekeeping gene. Normalized fold expression (nfe) of circRNAs was calculated by applying the 2^-ΔΔCt^ method. All the results were expressed as mean value of nfe ± SEM.

### PCR primer design

Primers to validate and amplify selected circRNAs in murine SPZ were designed through the online tool Primer-BLAST (http://www.ncbi.nlm.nih.gov/tools/primer-blast/). In order to make specific primers for the circular isoforms, we designed primers spanning the backsplicing junction. We also designed specific housekeeping *Cyclophilin* gene primers for normalization. Primer sequences are shown in **Table 2**.

**Table 2:** Primer Sequences and Annealing Temperatures.

Gene Primers	Sequences 5′-3′	Tm (°C)
*Mmu circ-MEMO1* S	ACTATGATGAATCCCAGGGGG	52
*Mmu circ-MEMO1* AS	CAGGGGCACATGATGGGAAG	
*Mmu circ-DNAH7* S	TACACGGGCCCTGCATTGTA	57
*Mmu circ-DNAH7* AS	AGGAGAGACCCAGCATGTGTA	
*Mmu circ-MAPT* S	GTCAGGTCGAAGATTGGCTCT	55
*Mmu circ-MAPT* AS	ATACTGGTTCAAAGCCTTGCC	
*Mmu circ-DNER* S	TGTGTCCTAGACCCATGCAG	53
*Mmu circ-DNER* AS	TCTGCAACAAACTTCCAGACAC	
*Mmu circ-CPSF6* S	TCGTTAGAAGATTTGCCCTTGT	52
*Mmu circ-CPSF6* AS	ACAACAGGACTCTGACCATGA	
*Mmu circ-PTPN11* S	TACGGGGTCATGCGTGTTAG	53
*Mmu circ-PTPN11* AS	GGGGTGAAACCATTTGTCCG	
*Mmu circ-ADAM10* S	CCTATGTCTTCACAGACCGGG	55
*Mmu circ-ADAM10* AS	TGGGGATAGTCTGAAGGTGC	
*Mmu circ-RESP18* S	TCTCCCCAAAAGATGGTCAGG	53
*Mmu circ-RESP18* AS	TGCCTTCGGGTACAATCTGG	
*Mmu circ-TULP4* S	ATAAACTTCAACCTGCGAGGC	53
*Mmu circ-TULP4* AS	TCCCGGTTAATTCAGGAGCCA	
*Cyclophilin-A* S	TGGTCTTTGGGAAGGTGAAAG	52
*Cyclophilin-A* AS	TGTCCACAGTCGGAAATGGT	

### Histology analysis

Testes and liver tissues collected from CTRL, HFD and FS mice (n=6 for each experimental groups) were fixed in Bouin’s solution overnight, dehydrated in ethanol, cleared in xylene, and embedded in paraffin using standard procedures. Tissue sections (7 µm thick) were deparaffined and stained with hematoxylin and eosin (H&E) reagents. *Cauda* SPZ collected from CTRL, HFD and FS mice (n=6 for each experimental group) were dried on slides and processed for H&E staining. Histological analyses were conducted under a light microscope (Leica CTR500, Leica Microsystems Inc., Milan, Italy) and images were captured using a high resolution digital camera (Leica DC300F). A minimum of 5 random sections was analyzed to evaluate hepatic fat vacuole accumulation in CTRL, HFD and FS mice and seminiferous epithelium morphology. For anomalous sperm head counts, a minimum of 100 sperm cells was counted for each assay and data were reported as the percentage of anomalous sperm head/total SPZ. As routinely required for these experimental procedures, all the results were validated twice by the same operator.

### Immunofluorescence analysis

SPZ collected from *caput/cauda* epididymis of CTRL, HFD and FS mice (n=4 for each experimental group) and *cauda* SPZ of CTRL mice *in vitro* treated with vehicle or H_2_O_2_ were used for immunofluorescence analysis. Samples were fixed in 4% paraformaldehyde (sc-281692; Santa Cruz Biotechnology, Heidelberg, Germany) for 20 min at RT. Permeabilization step was conducted with 0.1% Triton X-100 (X100; Sigma-Aldrich, Milano, Italy), while blocking was conducted with 10% of donkey serum (ab7475; Abcam, Cambridge, UK) for 30 min at RT. Slides were then incubated with different primary antibodies [anti-FUS antibody (PA5-52610; Invitrogen, Milano, Italy); anti-4HNE antibody (ab46545; Abcam, USA)] overnight at 4°C. Following three washes in Dulbecco’s PBS (DPBS, 1X), a conjugated secondary antibody was used (Jackson ImmunoResearch, Cambridge, UK) for 1 h at 37°C. Nuclei were labeled with DAPI (D9542; Sigma-Aldrich, Milano, Italy) and slides were observed under an optical microscope (Leica DM 5000 B + CTR 5000) with a UV lamp.

### Protein extraction and western blot analysis

*Caput* and/or *cauda* SPZ collected from CTRL, HFD and FS mice (n=6 animals for each experimental group) were used for total protein extraction. Samples were lysed in RIPA buffer [PBS, pH 7.4, 10 mM of dithiothreitol, 0.02% sodium azide, 0.1% SDS, 1% NP-40, and 0.5% sodium deoxycholate, protease inhibitors (10 μg/ml of leupeptin, aprotinin, pepstatin A, chymostatin, and 5 μg/ml of TPCK)] and sonicated three times for 30 sec bursts, each at 60 mW. Total lysates were separated by SDS-PAGE and transferred to polyvinylidene difluoride membrane (GE Healthcare, Milano, Italy) at 280 mA for 2.5 h at 4°C. The filters were treated for 3 h with blocking solution [5% nonfat milk, 0.25% Tween-20 in Tris-buffered saline (TBS, pH 7.6)] and then separately incubated with anti-FUS primary antibody (PA5-52610; Invitrogen, Milano, Italy) overnight at 4°C in TBS-milk buffer (TBS pH 7.6, 3% non-fat milk). After washing in 0.25% Tween20-TBS, filters were incubated with 1:1000 horseradish peroxidase-conjugated rabbit IgG (Dako Corp., Milano, Italy). The immune complexes were detected using the enhanced chemiluminescence-western blotting detection system [Amersham ECL western Blotting Detection Reagent (RPN2106) GE Healthcare, Milano, Italy]. The specificity of the immunoreactions was routinely checked by omitting primary antibody (data not shown).

### Functional annotation for circRNA/miRNA and target miRNA interaction

For circMEMO1 and circMAPT, the circRNA/miRNA interaction was predicted with Arraystar’s miRNA target prediction software, as previously reported (35). Validated or predicted targets of miRNAs were retrieved by Diana TarBase 8.0 (http://www.microrna.gr/tarbase); circRNA/miRNA/Target networks (ceRNETs) were built and visualized by using Bisogenet plug-in of Cytoscape (www.cytoscape.org).

### RNA binding protein immunoprecipitation assay (RIP)

For RIP assay, *caput* and *cauda* SPZ collected from CTRL, HFD and FS mice (n=6 samples for each experimental group) and *cauda* SPZ of CTRL mice *in vitro* treated with vehicle or H_2_O_2_ (n=6 samples for each experimental group) were lyzed in 500 µl of RIP lysis buffer (50 mM Tris-HCl pH 7.4; 150 mM NaCl; 5 mM EDTA; 1% NP-40; 0.1% SDS) supplemented with RNase inhibitors (100 U/ml) and protease inhibitors (10 μg/ml of leupeptin, aprotinin, pepstatin A, chymostatin, and 5 μg/ml of TPCK). A concentration of 500 µg of each lysate was incubated with 5 µg of FUS antibody (PA5-52610; Invitrogen, Milano, Italy) or IgG (12370; Sigma-Aldrich, Milano, Italy) under rotary agitation at 4°C overnight. Protein A/G PLUS Agarose Beads (sc-2003; Santa Cruz Biotechnology, Heidelberg, Germany) were added to each sample and incubated at 4°C for 4 h. Four washes with cold TBS pH 7.6, at 3000 × *g* for 5 min at 4°C, were conducted and then bead pellets were resuspended in 500 µl of Trizol Reagent (Invitrogen Life Technologies, Paisley, UK) to isolate RNA, following the manufacturer’s instructions. The immunoprecipitated RNAs with FUS and control IgG was quantized (ng/µl) using a NanoDrop 2000 spectrophotometer (Thermo, Waltham, MA, United States) and used for circMEMO1 and circMAPT qRT-PCR analysis, using specific primers.

### *In vitro* hydrogen peroxide treatment

*Cauda* epididymides collected from CTRL mice (n=6 for each experimental group) were separately immersed in PBS (pH 7.6) and cut to let SPZ flow out from the ducts. SPZ were then purified and collected as above reported. SPZ pellets (1 x 10^7^ cells for each experimental group) were separately treated with *vehicle* (PBS pH 7.6) or H_2_O_2_ (Sigma, USA) at the final concentration of 200 μM as previously reported (38). Following incubation at 37°C for 2 h, aliquots of samples were used for sperm motility analysis, dried on slides to be stored at -20°C for immunofluorescence analyses and/or stored at -80°C for molecular investigations as above reported.

### Statistical analysis

Student’s t-test (for two independent group comparison) and ANOVA followed by Duncan’s test (for multi group comparison) were conducted to identify groups with a different mean. Data were expressed as the mean ± SEM from at least 6 independent animals for each experimental group. For qRT-PCR and western blot analyses, triplicates from each 6 animals/experimental group were considered.

## Results

### Impact of FS treatment on HFD phenotype and reproductive parameters

**Figure 1:**
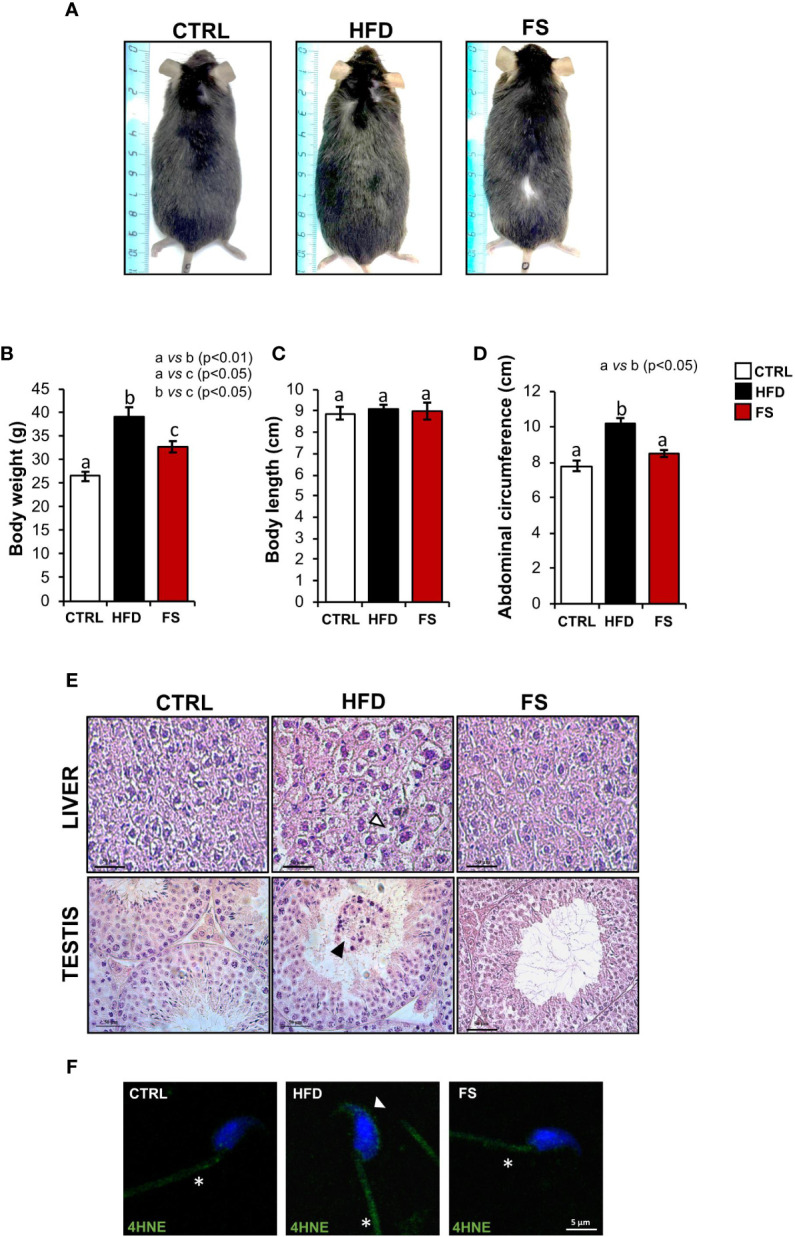
Effects of food supplementation (FS) administration on body parameters. **(A)** Representative image of CTRL, HFD and FS (HFD +FS) male mice. **(B–D)** Body weight **(B)**, body length **(C)** and abdominal circumference **(D)** in CTRL (n=12), HFD (n=12) and FS (n=12) mice. All data are reported as mean ± SEM. Different letters indicate statistical significance (p<0.05 or p<0.01). **(E)** Hematoxylin and eosin (H&E) staining of Bouin’s fixed CTRL, HFD and FS liver and testis sections (7 μm). Fat vacuole accumulation in hepatic cell was indicated by white arrowheads while in testis, detached germ cells were indicated by black arrowheads. Scale bar: 50 μm. **(F)** Immunofluorescence analysis of 4-Hydroxy-2-nonenal (4HNE) in *cauda* SPZ of CTRL, HFD and FS mice. White arrowheads and white asterisk represent 4HNE localization (green) in sperm head and tail, respectively. Nuclei were labeled with DAPI (blue). Scale bar: 5 µm.

*In vivo* treatment with FS was carried out in HFD (60% fat) C57BL/6 male mice model for 4 weeks to investigate a potential effect on body mass and lipid parameter restoration to physiological condition. After the treatment, the mice size was reduced in comparison with HFD mice appearing similar to CTRL ones (**Figure 1A**). A significant reduction of body weight (p<0.01), not completely restored to CTRL values, was also observed in FS as compared with HFD mice (**Figure 1B**), while no significant changes were observed in the body length (**Figure 1C**). Instead, a significant reduction of abdominal circumference (p<0.01) to physiological values occurred in FS treatment group (**Figure 1D**). In order to confirm FS action on lipid metabolism, liver morphology was assessed by H&E staining. As showed, the typical hepatic steatosis and fat vacuole accumulation in the hepatic cells, occurring in HFD condition, were completely reverted in FS experimental group (**Figure 1E**). In agreement, a significant (p<0.01) reduction of HFD liver weight (1.62 ± 0.03) to CTRL values (1.30 ± 0.05) occurred in FS experimental group (1.29 ± 0.06) (**Table 3**).

**Table 3:** Effects of FS treatment on liver weight and lipid concentrations.

Liver (g)		TG (mmol/L)	TC (mmol/L)	HDL (mmol/L)	LDL (mmol/L)
1.30 ± 0.05	CTRL	0.92 ± 0.04	1.85 ± 0.17	1.25 ± 0.09	0.59 ± 0.04
1.62** ± 0.03	HFD	12.89 ± 0.05**	4.89 ± 0.12**	4.23 ± 0.11**	1.28 ± 0.08*
1.29 ± 0.06	FS	0.98 ± 0.12	1.97 ± 0.13	1.20 ± 0.10	0.62 ± 0.07

Data are expressed relatively to the CTRL group and reported as mean ± SEM; *p<0.05; **p<0.01.

Considering that in our previous work we have demonstrated an impaired testicular morphology and several spermatic anomalies, oxidative stress dependent in HFD male mice (35), we assessed a potential beneficial effect of FS on HFD reproductive phenotype. In detail, we analyzed testis morphology and sperm oxidative state by H&E staining and 4HNE immunofluorescence analysis, respectively. Confirming what we have previously demonstrated, HFD mice showed an impaired testicular morphology characterized by a seminiferous epithelium disorganization and a germ cell detachment, thus suggesting a functional injury of the blood - testis - barrier. Interestingly, this anomalous phenotype was reverted in FS experimental group (**Figure 1E**). Then, we carried out the immunofluorescence analysis of 4HNE, known to inactivate some antioxidant enzymes and affect mitochondria by impairing ATPase activity (39, 40). Our results demonstrated a strong 4HNE signal in both HFD sperm head and tail, confirming a state of oxidative stress, that significantly reduced in FS SPZ (**Figure 1F**).

### FS treatment restored spermatic circRNA cargo in HFD mice

**Figure 2:**
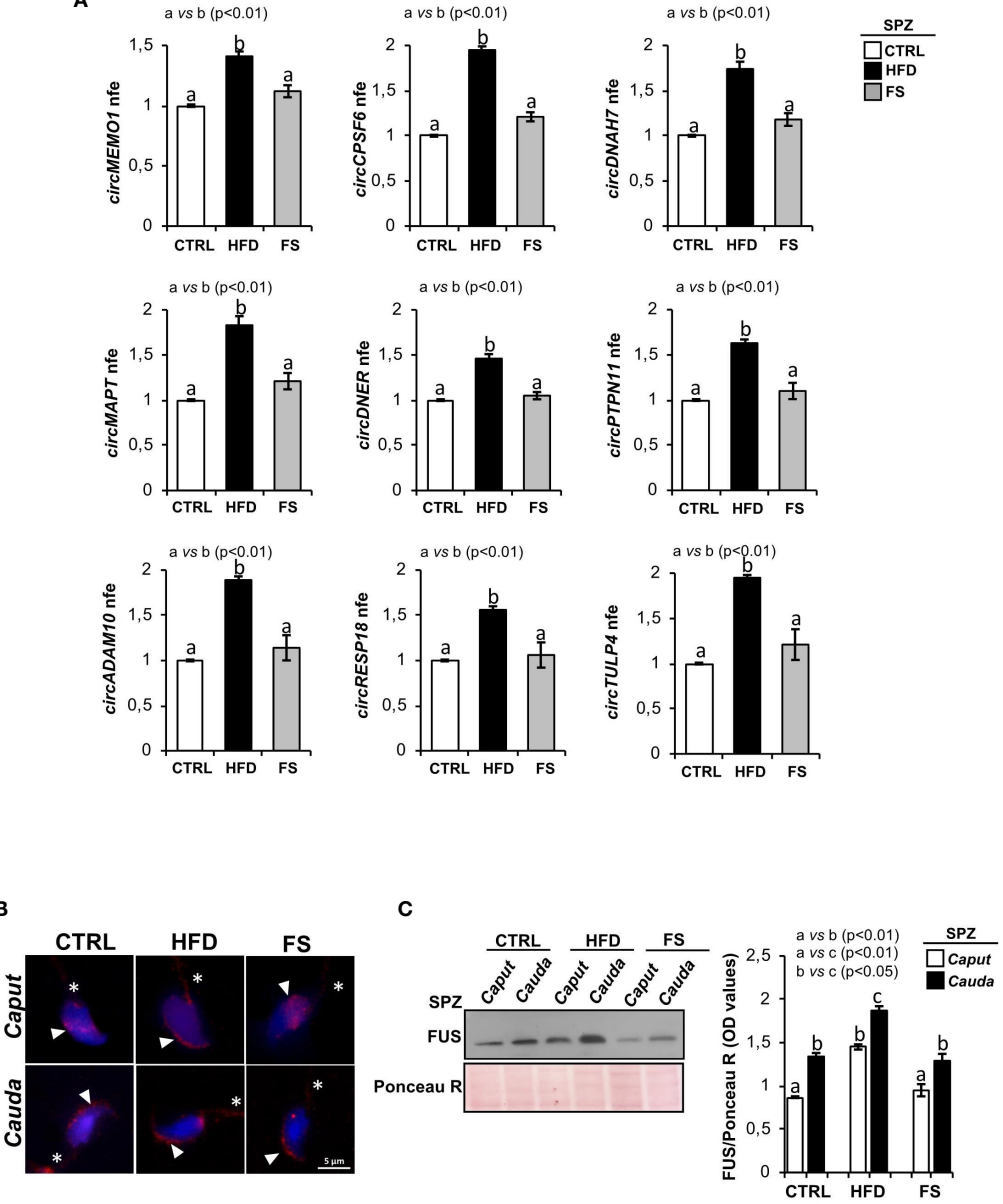
Effects of food supplementation (FS) administration on circRNA and FUS expression in SPZ. **(A)** Expression analysis of 9 circRNAs up-regulated in CTRL, HFD and FS sperm cells. qRT-PCR data are normalized using *Cyclophilin* expressed as fold expression (nfe) and reported as mean value ± S.E.M. a *vs* b: p< 0.01. **(B)** Immunofluorescence analysis of FUS protein in *caput* and *cauda* SPZ of CTRL, HFD and FS mice. White arrowheads and white asterisk represent FUS localization (red) in sperm head and tail, respectively. Nuclei were labeled with DAPI (blue). Scale bar: 5 µm. **(C)** Western blot analysis of FUS protein in *caput* and *cauda* SPZ of CTRL, HFD and FS mice (n=6 different samples for each experimental group in triplicate). Signals were quantified by densitometry analysis and normalized to Ponceau Red (Ponceau R). Data were expressed in OD values and reported as mean ± SEM. Different letters indicate statistical significance (p<0.05 or p<0.01).

We have previously characterized a spermatic circRNA cargo in HFD SPZ, focusing on a set of differentially expressed circRNAs (DE-circRNAs). An enhanced endogenous backsplicing in sperm cells was the driving force for circRNAs up-regulated in HFD SPZ and potentially involved in the oxidative stress pathway (35). With this wealth of knowledge, in the current work, we aimed to assess the potential FS ability in restoring a physiological spermatic circRNA cargo. In detail, epididymal SPZ collected from CTRL, HFD and FS mice were used to analyze the expression levels of 9 selected circRNAs up-regulated in HFD SPZ, as previously identified (35). In agreement with our data, all the analyzed circRNAs were significantly (p<0.01) up-regulated in HFD SPZ (**Figure 2A**). Interestingly, FS treatment restored circRNA expression levels to physiological values.

In order to clarify the molecular mechanism underlying the resetting of circRNA profile in HFD SPZ following FS treatment, we pointed on FUS protein, the main RBP orchestrating spermatic circRNA biogenesis (34) and previously reported as key player in the enhanced endogenous backsplicing in HFD SPZ (35). We carried out a qualitative and quantitative FUS characterization in SPZ collected from *caput* and *cauda* epididymis of CTRL, HFD and FS mice, by immunofluorescence and western blot analyses, respectively. Accordingly to our previous data (35), immunofluorescence analysis confirmed in *caput* HFD SPZ an early FUS acquisition in periacrosomal region, typically observed in *cauda* CTRL SPZ. Interestingly, in FS group, a regular pattern of spermatic FUS distribution from *caput* to *cauda*, comparable with CTRL one, was observed (**Figure 2B**). Western blot analysis showed a significant increase of FUS content (p<0.01) from *caput* to *cauda* in all the investigated experimental groups (**Figure 2C**). However, a higher FUS content, comparable with *cauda* CTRL SPZ levels, was observed in *caput* HFD SPZ (**Figure 2C**). Once again, FS treatment restored FUS content to physiological values in both *caput* and *cauda* SPZ (**Figure 2C**).

### FS treatment restored spermatic backsplicing mechanism and functional parameters of HFD SPZ

Considering that HFD condition induced spermatic oxidative stress affecting the endogenous backsplicing activity (35), we shed light on the potential effect of FS treatment in restoring sperm functional parameters, *via* oxidative state - backsplicing axis modulation.

**Figure 3:**
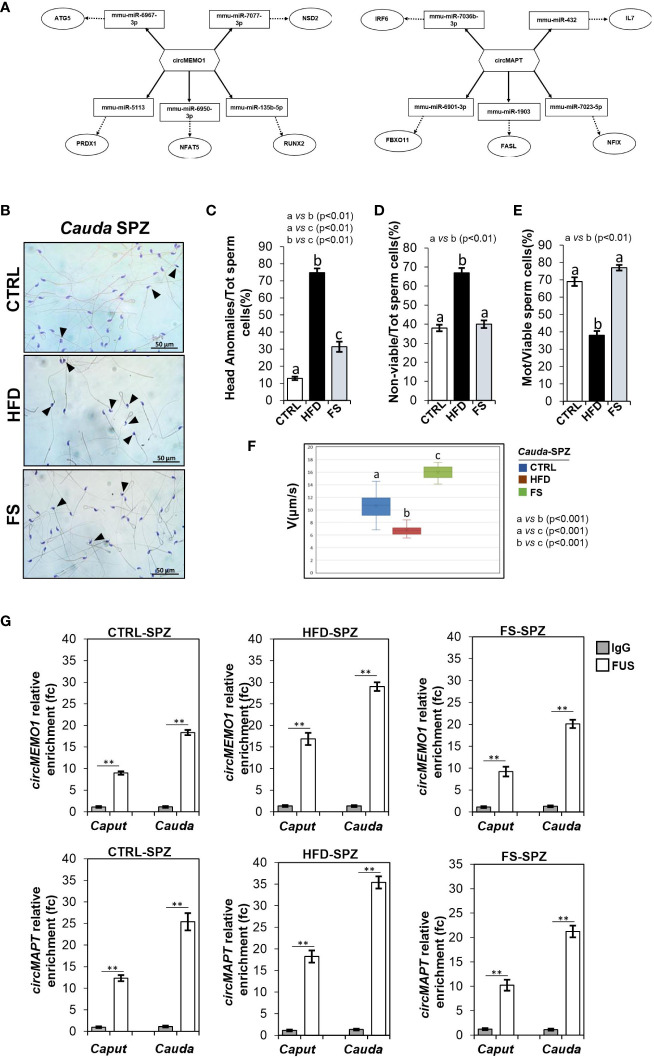
Effects of food supplementation (FS) administration on sperm backsplicing machinery and functional parameters. **(A)** Two circRNAs up-regulated in HFD SPZ, circMEMO1 and circMAPT, tether a group of miRNAs and mRNAs as targets, all involved in oxidative stress and motility pathways. Networks were built using Cytoscape. Hexagonal and rectangular symbols represent circRNAs and miRNAs, respectively. The arrow indicates the tethering activity of circRNAs toward miRNAs, while the dotted arrow indicates the pathways downstream of the miRNAs. **(B)** Hematoxylin and eosin (H&E) staining of *cauda* SPZ collected from CTRL (n=5), HFD (n=5) and FS (n=5) mice; anomalous sperm heads were indicated by black arrowheads. Scale bar: 50 μm. **(C)** Percentage of anomalous sperm head in CTRL, HFD and FS *cauda* SPZ; data were reported as the percentage of anomalous sperm head/total SPZ. Sperm death **(D)** and motility **(E)** assay in CTRL, HFD and FS *cauda* SPZ; data were expressed as the percentage of non-viable/total SPZ and motile/live SPZ, respectively and reported as mean ± SEM. Different letters indicate statistical significance (p<0.01). **(F)** Sperm motility evaluation in CTRL, HFD and FS *cauda* SPZ by TLVM analysis. Sperm velocity was calculated in µm/s; different letters indicate statistical significance (p<0.001). **(G)** The enrichment levels of circMEMO1 and circMAPT in the products of RNA binding protein immunoprecipitation (RIP) assay (FUS-IP compared with IgG-IP) in *caput* and *cauda* SPZ of CTRL, HFD and FS mice detected by qRT-PCR. Data were reported as mean ± SEM from three independent experiments. **p<0.01.

To achieve our goal, by using a bioinformatic approach, we built ceRNETs for two circRNAs up-regulated in HFD SPZ, potentially responsive to oxidative stress, in order to identify the mRNA targets involved in the regulation of sperm functional parameters dependent on the oxidative state. Among circRNAs up-regulated in HFD SPZ we chose circMEMO1, because it’s *in vitro* silencing promoted cell viability by the reduction of oxidative stress (41), and circMAPT because mutations in its linear counterpart increase mitochondrial membrane potential, leading in turn to reactive oxygen species (ROS) production, oxidative stress and neuronal death (42) (**Figure 3A**). Bioinformatic analyses provided the top five miRNA targets, preferentially involved in oxidative stress and sperm motility, as above reported. In detail, miRNAs identified for circMEMO1 were: mmu-miR-6967-3p; mmu-miR-7077-3p; mmu-miR-513; mmu-miR-6950-3p; mmu-miR-135b-5p and for circMAPT were: mmu-miR-7036b-3p; mmu-miR-432; mmu-miR-6901-3p; mmu-miR-1903; mmu-miR-7023-5p (**Figure 3A**).

H&E staining was carried out in SPZ collected from *cauda* epididymis, which represent the spermatic population having the fertilizing ability (**Figure 3B**). As showed, in FS group a significant reduction (p<0.01) in the number of SPZ with structural defects in sperm head occurred (**Figure 3C**), however not at physiological levels, suggesting that FS treatment restored spermatic HFD dependent anomalies. In agreement, sperm functional parameters, as viability and motility, were also investigated. As reported, a significant reduction in the percentage of non-viable sperm cells (p<0.01) was observed in FS group as compared to CTRL one (**Figure 3D**), whereas the drastic reduction of motility observed in HFD mice was significantly (p<0.01) improved to physiological values following FS treatment (**Figure 3E**), confirming that FS potentially reverted the oxidative state and, in turn, sperm motility. Consistently, sperm motility was also quantitatively investigated by TLVM analysis that allowed us to specifically calculate sperm cell velocity. The graph shows the uniformity of distribution since median value overlaps with medium one, confirming that sperm motility analysis occurred in a suitable time interval to obtain relevant outcomes. The video microscopy station and the fine thermal control are relevant to the robustness of analyses. As reported in **Figure 3F**, a significant reduction (p<0.001) of sperm motility was observed in HFD when compared to CTRL SPZ. FS treatment induced a significant 1.6 and 2.4-fold increase of sperm motility in comparison with CTRL and HFD experimental groups (**Figure 3F**), respectively, confirming that FS treatment positively modulated sperm motility.

To demonstrate that the improvement of HFD sperm motility, occurring following FS treatment, may be related to the recovery of circRNA profile, *via* the regulation of FUS dependent endogenous backsplicing machinery, we carried out a RIP assay in *caput* and *cauda* SPZ of CTRL, HFD and FS mice, using FUS antibody. In detail, we aimed to evaluate if the restoration of FUS levels observed in FS SPZ could correlate with a fine regulated backsplicing activity responsible for the up-regulated DE-circRNA recovery. CircMEMO1 and circMAPT were chosen for this investigation as representative circRNAs up-regulated in HFD SPZ, predicted to be responsive to oxidative stress pathway and involved in sperm motility modulation. Relatively to the use of control IgG, RIP results showed a significant 8.97- and 18.13-fold enrichment of circMEMO1 (p<0.01) in *caput* and *cauda* CTRL SPZ, respectively (**Figure 3G**). Confirming the enhanced endogenous backsplicing skill of HFD SPZ, a significant 16.87- and 28.98-fold enrichment of circMEMO1 (p<0.01), greater than what observed in CTRL SPZ, occurred in *caput* and *cauda* HFD SPZ (**Figure 3G**). Interestingly, RIP assay carried out in FS SPZ showed similar values observed for CTRL ones, as a significant 9.23- and 20.12-fold enrichment of circMEMO1 (p<0.01) occurred in *caput* and *cauda* SPZ, respectively, confirming that FS treatment restored the regular endogenous backsplicing (**Figure 3G**). Similarly, RIP assay for circMAPT showed the same profile of circMEMO1. In detail, a significant 12.34- and 25.4-fold enrichment of circMAPT (p<0.01) was observed in *caput* and *cauda* CTRL SPZ, respectively (**Figure 3G**). Once again, HFD SPZ showed an increased backsplicing activity; indeed, a significant 18.23- and 35.4-fold enrichment of circMAPT (p<0.01), higher than CTRL ones, was observed in *caput* and *cauda* HFD SPZ. As expected, a fold enrichment of 10.23 and 21.23, relatively to *caput* and *cauda* FS SPZ, respectively, occurred following FS treatment, confirming a restored spermatic backsplicing activity likely dependent on the oxidative state (**Figure 3G**).

### *In vitro* assessment of oxidative stress - circRNAs - sperm quality functional axis

We hypothesized that sperm oxidative state modulates circRNA backsplicing and in turn putative ceRNETs involved in the regulation of sperm motility. To confirm this hypothesis, we set a key experimental strategy. In detail, *cauda* SPZ collected from CTRL mice were *in vitro* treated with vehicle (*Ctrl-*group) or hydrogen peroxide (H_2_O_2_) (*Exp-*group), widely used to induce oxidative stress in cellular models (43), at the concentration of 200 μM, reported to be the optimal dose to affect sperm motility (38).

**Figure 4:**
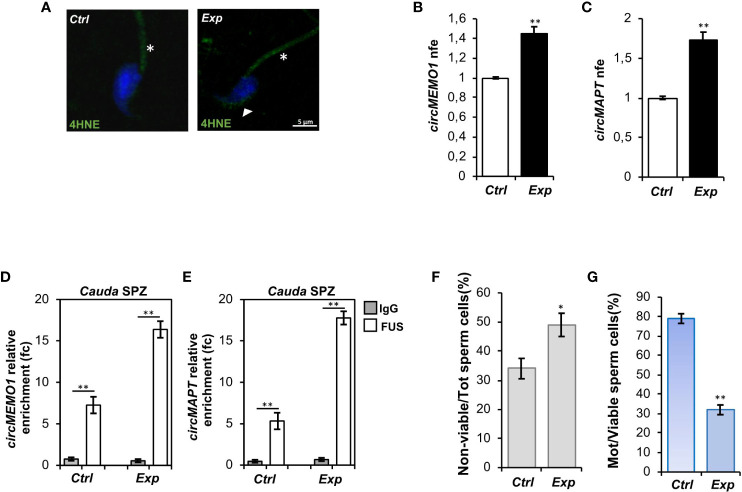
Effects of H_2_O_2_ *in vitro* treatment on oxidative stress, circRNA expression and sperm functional parameters. **(A)** Immunofluorescence analysis of 4-Hydroxy-2-nonenal (4HNE) in *cauda* SPZ *in vitro* treated with vehicle (*Ctrl*) or H_2_O_2_ (*Exp*). White arrowheads and white asterisk represent 4HNE localization (green) in sperm head and tail, respectively. Nuclei were labeled with DAPI (blue). Scale bar: 5 µm. **(B, C)** Expression analysis of circMEMO1 **(B)** and circMAPT **(C)** in *Ctrl* and *Exp cauda* SPZ. qRT-PCR data are normalized using *Cyclophilin* expressed as fold expression (nfe) and reported as mean value ± S.E.M; **p<0.01. **(D, E)** The enrichment levels of circMEMO1 and circMAPT in the products of RNA binding protein immunoprecipitation (RIP) assay (FUS-IP compared with IgG-IP) in *Ctrl* and *Exp cauda* SPZ by qRT-PCR. Data are reported as mean ± SEM from three independent experiments; **p<0.01. **(F, G)** Sperm death **(F)** and motility **(G)** assay in *Ctrl* and *Exp cauda* SPZ; data were expressed as the percentage of non-viable/total SPZ and motile/live SPZ, respectively and reported as mean ± SEM; *p<0.05; **p<0.01.

Immunofluorescence analysis of 4HNE was carried out on both experimental groups. As observed, a high 4HNE signal intensity occurred following H_2_O_2_ *in vitro* treatment, confirming lipid peroxidation increase and thus oxidative stress onset (**Figure 4A**). To demonstrate that the experimental induction of oxidative stress affected spermatic backsplicing activity, we investigated the expression of circMEMO1 and circMAPT by qRT-PCR analysis. Relatively to *Ctrl* group, both circRNAs were significantly up-regulated (p<0.01) following H_2_O_2_ *in vitro* treatment (**Figures 4B, C**), confirming our hypothesis. In order to assess whether the increase of circMEMO1 and circMAPT was dependent on the enhanced FUS activity, we carried out a RIP assay in *cauda Ctrl* and *Exp* SPZ by using FUS antibody. Relatively to the use of control IgG, RIP results showed a significant 7.27- and 16.38-fold enrichment of circMEMO1 (p<0.01) in *Ctrl* and *Exp* SPZ, respectively (**Figure 4D**). Similarly, RIP assay for circMAPT showed a significant 5.32- and 17.78-fold enrichment (p<0.01) relatively to control IgG, in *Ctrl* and *Exp* SPZ, respectively (**Figure 4E**), confirming the triggered FUS dependent backsplicing machinery following *in vitro* induction of oxidative stress.

Finally, sperm viability and motility were also investigated. As reported, a significant increase (p<0.05) in the percentage of non-viable sperm cells (**Figure 4F**), in addition to a significant reduction (p<0.01) of sperm motility (**Figure 4G**), were observed in *Exp* as compared with *Ctrl* SPZ, demonstrating a functional interplay among oxidative stress, circRNAs and sperm motility.

## Discussion

Changes in the lifestyle of civilized countries are strongly related to bad eating habits. Obesity and related dysfunctions, among them male infertility, are a dramatic consequence (44–46). The decline of sperm quality during the obesity onset is often established by oxidative stress. Indeed, oxidative stress activates an intrinsic macrophage response with consequent sperm DNA damage, sperm motility reduction and embryo implantation failure (46–48). In this scenario, antioxidant administration proved a valid approach to improve male fertility. The efficacy of vitamin E and C on male infertility has been established in supporting sperm functional parameters and, in turn, pregnancy success (49). Interestingly, vitamin E treatment combined with selenium, a powerful antioxidant agent that protects cellular membranes from peroxidative damage, improves sperm motility in asthenoteratozoospermic patients (50), while vitamin C supplementation in infertile men supports sperm count and morphology, favoring semen quality towards conception (25). Fish oil, with a selected composition (e.g. ω-3 ω-6), has been used for more than a decade to counteract lipid deposits, with specific reference to LDL (low density lipoprotein) cholesterol or to HDL (high density lipoprotein) on LDL cholesterol ratio (HDL/LDL), thus to prevent cardiovascular diseases (51, 52). Here these components, added to the vitamins and selenomethionine, may further support the whole lipid metabolism to diminish the well-known inflammatory detrimental status strictly linked to the fat deposit in overweight subjects. Beside this, it is important to evaluate the biochemical and biological bases characterizing the pathological condition and/or reproductive dysfunction. To this end, the study of new biomarkers useful for sperm quality setting is continuously developing in the reproductive field. In this context, circRNAs, consisting of non-coding RNA molecules involved in post-transcriptional regulation of gene expression, are acquiring a great interest as they have been well characterized in human SPZ in terms of cellular localization and molecular functions (30). In addition, circRNA deregulation occurs in pathological conditions, including asthenozoospermia (31) and affects nuclear architecture during sperm epididymal maturation (32). Previously, we have analyzed a set of DE-circRNAs, influenced by HFD condition and produced by an enhanced FUS dependent backsplicing (35). Several ceRNETs, potentially impacting sperm functions *via* oxidative stress, have also been predicted. Based on these preliminary data, in the current work, we provide a new exciting interplay between circRNAs and sperm functions, negatively affected by metabolic dysfunctions. To this end, we used HFD male mice, orally treated with a dietary supplemental mix of vitamins C-E-B12, GSH and selenium-L-methionine, as an antioxidant dependent metabolic restoration model. As expected, antioxidant administration significantly attenuated HFD phenotype by reducing body weight and abdominal circumference as well as serum lipid levels and liver steatosis. Considering that: i) low vitamin B12 levels have been associated with obesity and overweight; ii) vitamin C intake may help in preventing obesity onset in humans and animals; iii) metabolic syndrome condition is attenuated by vitamin E, our data are consistent (17, 19, 53).

In terms of circRNA cargo, we assessed its potential modulation by antioxidants. Interestingly, all circRNAs, up-regulated in HFD SPZ, were totally restored to physiological values by antioxidants. In this respect, to focus on FUS as a main actor in sperm circRNA biogenesis was a good strategy. In detail, FUS protein increased in HFD SPZ and early acquired a cellular localization typically observed during a physiological epididymal transit, as previously demonstrated (30, 35); antioxidants reverted such a profile, highlighting, for the first time, an effect of the sperm oxidative state on backsplicing. Thinking about the existence of a potential functional axis among oxidative stress - circRNAs - sperm quality, we chose to analyze circMEMO1 and circMAPT dependent networks because both involved in oxidative stress response (41, 42). Through specific miRNAs, circMEMO1 controls the expression of several targets involved in the oxidative stress, as: i) ATG5, a key factor in both basal and drug-induced oxidative stress, also required for elongating spermatid development (54, 55); ii) WNT1, a key factor in the modulation of mitochondrial membrane permeability and apoptotic cascade dependent on oxidative stress (56); iii) PRDX1, an antioxidant enzyme associated with lipid peroxidation and decreased motility of epididymal SPZ (57). Similarly, circMAPT regulates the expression of: i) IRF6, whose low levels reduce ROS accumulation to preserve mitochondrial function (58); ii) FXBO11, responsible for oxidative stress regulation in cell lines (59).

In light of this, we demonstrated that the antioxidant treatment improved sperm morphological and functional anomalies associated with HFD dependent oxidative stress, by reducing sperm head defects, increasing sperm viability and motility. The application of TLVM further corroborated data related to sperm motility, suggesting that this quantitative approach, with the specific cell trucker software, may help in better understanding real time biological features in diverse experimental groups. Furthermore, the antioxidant treatment restored sperm physiological oxidative state as indicated by 4HNE reduction, an aldehyde product derived from lipid peroxidation, preferentially chosen to diagnose oxidative stress in the male germ cells (60). In addition, a proper FUS dependent sperm backsplicing, confirmed by circMEMO1 and circMAPT RIP experiments, was recovered, corroborating our hypothesis. Evidences collected from the *in vitro* H_2_O_2_ treatment of *cauda* SPZ definitively strengthened the existence of a functional axis. Indeed, the induction of oxidative stress, confirmed by the increase of 4HNE marker, not only enhanced FUS dependent backsplicing of circMEMO1 and circMAPT, but also negatively affected sperm motility.

Collectively, our data highlight a new fascinating molecular axis in which the oxidative stress - triggered in metabolic dysfunctions - switches on the endogenous sperm backsplicing machinery that, in turn, promotes selective ceRNETs, downstream regulating sperm motility. Our work also confirms HFD dependent effects on sperm circRNAs, clarifying their role in sperm oxidative stress and motility anomalies. In addition, the beneficial antioxidant action not only on sperm oxidative state, but also on circRNA production and sperm quality was highlighted.

## Data availability statement

The datasets in this study are available from the corresponding author upon reasonable request.

## Ethics statement

The animal study was approved by Italian Ministry of Education and the Italian Ministry of Health. The study was conducted in accordance with the local legislation and institutional requirements.

## Author contributions

VM: Investigation, Methodology, Writing – original draft. TC: Formal Analysis, Methodology, Writing – original draft. RF: Methodology, Writing – original draft. AD’A: Investigation, Methodology, Writing – original draft. Md’A: Investigation, Methodology, Writing – original draft. DC: Formal Analysis, Methodology, Writing – original draft. MM: Formal Analysis, Writing – original draft. VP: Investigation, Methodology, Writing – original draft. AG: Resources, Writing – review & editing, Visualization. SF: Visualization, Writing – review & editing. GC: Visualization, Writing – review & editing. CS: Conceptualization, Funding acquisition, Supervision, Writing – review & editing. RC: Conceptualization, Supervision, Writing – review & editing. FM: Conceptualization, Formal Analysis, Investigation, Methodology, Writing – original draft.
